# Accuracy of non-invasive cuffless blood pressure in the intensive care unit: Promises and challenges

**DOI:** 10.3389/fmed.2023.1154041

**Published:** 2023-04-17

**Authors:** Sondre Heimark, Kasper Gade Bøtker-Rasmussen, Alexey Stepanov, Øyvind Gløersen Haga, Victor Gonzalez, Trine M. Seeberg, Fadl Elmula M. Fadl Elmula, Bård Waldum-Grevbo

**Affiliations:** ^1^Department of Nephrology, Oslo University Hospital, Ullevål, Oslo, Norway; ^2^Institute of Clinical Medicine, University of Oslo, Oslo, Norway; ^3^Aidee Health AS, Oslo, Norway; ^4^Department of Smart Sensors and Microsystems, SINTEF Digital, Oslo, Norway; ^5^Cardiorenal Research Centre, Oslo University Hospital, Ullevål, Oslo, Norway

**Keywords:** cuffless, blood pressure, pulse arrival time, machine learning, intensive care unit

## Abstract

**Objective:**

Continuous non-invasive cuffless blood pressure (BP) monitoring may reduce adverse outcomes in hospitalized patients if accuracy is approved. We aimed to investigate accuracy of two different BP prediction models in critically ill intensive care unit (ICU) patients, using a prototype cuffless BP device based on electrocardiogram and photoplethysmography signals. We compared a pulse arrival time (PAT)-based BP model (generalized PAT-based model) derived from a general population cohort to more complex and individualized models (complex individualized models) utilizing other features of the BP sensor signals.

**Methods:**

Patients admitted to an ICU with indication of invasive BP monitoring were included. The first half of each patient’s data was used to train a subject-specific machine learning model (complex individualized models). The second half was used to estimate BP and test accuracy of both the generalized PAT-based model and the complex individualized models. A total of 7,327 measurements of 15 s epochs were included in pairwise comparisons across 25 patients.

**Results:**

The generalized PAT-based model achieved a mean absolute error (SD of errors) of 7.6 (7.2) mmHg, 3.3 (3.1) mmHg and 4.6 (4.4) mmHg for systolic BP, diastolic BP and mean arterial pressure (MAP) respectively. Corresponding results for the complex individualized model were 6.5 (6.7) mmHg, 3.1 (3.0) mmHg and 4.0 (4.0) mmHg. Percentage of absolute errors within 10 mmHg for the generalized model were 77.6, 96.2, and 89.6% for systolic BP, diastolic BP and MAP, respectively. Corresponding results for the individualized model were 83.8, 96.2, and 94.2%. Accuracy was significantly improved when comparing the complex individualized models to the generalized PAT-based model in systolic BP and MAP, but not diastolic BP.

**Conclusion:**

A generalized PAT-based model, developed from a different population was not able to accurately track BP changes in critically ill ICU patients. Individually fitted models utilizing other cuffless BP sensor signals significantly improved accuracy, indicating that cuffless BP can be measured non-invasively, but the challenge toward generalizable models remains for future research to resolve.

## 1. Introduction

At present, blood pressure (BP) monitoring in hospitalized patients is limited to either intermittent cuff-based measurements or invasive arterial catheterization. Invasive arterial BP monitoring is the only method capable of accurate in-hospital continuous BP monitoring and is considered the gold standard given correct operating conditions. However, it is only available during surgery, post-operatively or in intensive care units (ICU) and requires specialized personnel. In addition, arterial catheterization carries risk such as bleeding, arterial occlusion and infection. For the remainder of hospitalized patients, BP is taken intermittently at varying intervals. Undetected hypotensive episodes may lead to organ damage such as acute kidney injury, and undetected clinical deterioration may delay adequate treatment and lead to adverse outcomes (1, 2). Studies indicate that adverse events are related to the intermittent nature of vital signs monitoring on hospital wards (3, 4). Thus, there is a clear need for non-invasive continuous cuffless BP monitoring in hospitalized patients to bridge the gap between intermittent cuff-based measurements and invasive arterial catheterization.

Despite substantial research on methods to enable non-invasive cuffless BP monitoring, its general accuracy remains uncertain, and few studies have investigated accuracy in critically ill patients. In addition, non-invasive cuffless BP methods use different approaches such as pulse wave propagation-based measurements (such as pulse arrival time (PAT)) and photo-plethysmography (PPG) waveform features. Studies, including research performed by our multidisciplinary team, have shown strong correlations between PAT and BP, particularly during various exercise methods (5–9) but its accuracy across differing populations and hemodynamic conditions are uncertain (6). New advances in non-invasive cuffless BP indicate that complex modeling by machine learning methods of sensor-based measurements are key toward improved results (6). In the present study, we aimed to investigate accuracy of two different BP-prediction models using the signals from a prototype chest belt BP sensor in critically ill patients. Specifically, we investigated a PAT-based model, derived from a general population cohort (generalized PAT-based model) compared to continuous invasive BP measurements and compared it with accuracy of individually fitted machine learning models (complex individualized models) that utilized other features of the signals obtained by the cuffless BP sensor.

## 2. Materials and methods

### 2.1. Subjects

Patients older than 18 years admitted to the general medical ICU at Oslo University Hospital, Ullevål were considered for inclusion. Inclusion criteria were signed consent and an inserted arterial line. Exclusion criteria were ongoing arrythmias generating irregular R-R intervals, failure to obtain adequate signals from the cuffless device or any medical contraindication to having a chest belt mounted. Each patient was monitored for a duration of 1–12 h, depending on length of stay, discontinuation of the intra-arterial catheter or other clinical interruptions.

### 2.2. Reference blood pressure

Reference BP was measured continuously with a radial artery catheter connected by a fluid filled tube to a pressure transducer (Xtrans; Codan, Forstinning, Germany). The pressure transducer was leveled at the phlebostatic axis and had a saline flush connected with a counterpressure of approximately 300 mmHg. The system was connected to a Philips IntelliVue MX 800 patient monitor (Philips, Böblingen, Germany). Zeroing was performed every 8-h according to the ICUs procedures. All vital signs, including the raw arterial waveform and the monitor-generated absolute BP values sampled every 5 s, were recorded directly to a laptop *via* an RS-232 connection using the Vital Recorder software (10).

### 2.3. Cuffless blood pressure device

**Figure 1:**
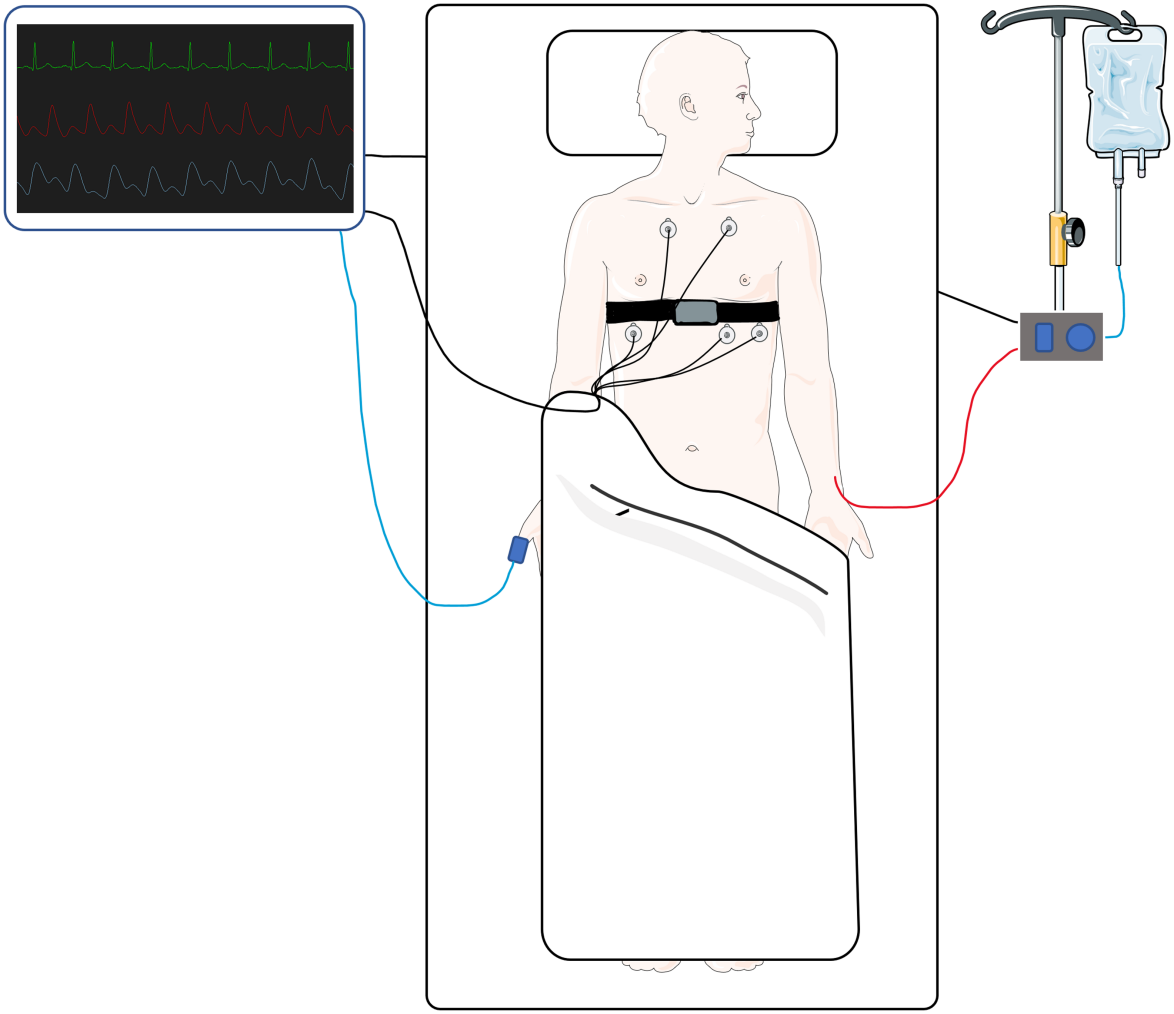
A simplified illustration of the chest belt device (cuffless device) fitted on a patient in the intensive care unit alongside basic monitoring equipment. Parts of the figure were created by using pictures from Servier Medical Art. Servier Medical Art by Servier is licensed under a Creative Commons Attribution 3.0 Unported License (https://creativecommons.org/licenses/by/3.0/).

A prototype cuffless BP sensor (cuffless BP device) was used in this study (7–9). It consists of a one-channel electrocardiogram (ECG) sensor, a photo-plethysmography (PPG) sensor and an inertial measurement unit (3D accelerometer and 3D gyroscope) integrated in a wearable chest belt. Raw signals from the ECG and PPG sensors were sampled at 1,000 Hz, while accelerometer data was sampled at 208 Hz and gyroscope data that were sampled at 26 Hz. The gyroscope data was not used. The cuffless BP device was fitted as illustrated in Figure 1. The generalized PAT-based model was developed from BP changes during isometric exercise in a general population cohort (9), using PAT and HR as cuffless surrogates but not any demographic information. A linear best fit equation with a coefficient for PAT, a coefficient for interaction between PAT and HR (this term was negligible) and a coefficient for HR was used. Additionally, we computed a best fit linear model using only PAT. The complex individualized models, utilizing other signal features, were trained using the first half of each patient’s data. Thus, the test period for both models were defined as the second half of each patient’s data. The cuffless BP device was calibrated against the first three minutes of reference BP at the start of each test period. This was a simple static calibration to correct the offset between average reference BP and cuffless BP across the initial three minutes. Since the pressure transducer was mounted on a bracket next to the patient bed, temporary periods occurred of which the pressure transducer moved relative to the phlebostatic axis. To reliably exclude such periods, an investigator continuously observed all data collections. In addition, if the pressure transducer moved significantly during such a period and was relevelled by the ICU staff, the cuffless BP device was re-calibrated against reference BP during the test period. Recalibration occurred in 14 patients (once in seven patients, twice in four patients and three times in two patients). Reasons for recalibration were related to nursing care, changing from supine bed rest to seated position or temporary detachment from the invasive monitoring system because of imaging studies or bathroom visits. Recalibration was decided necessary to avoid systematic biases introduced during relevelling. For example, if the pressure transducer was relevelled one time during a patient’s data collection with an offset of 5 cm relative to the previous leveling, a systematic bias of 3.7 mmHg would be introduced for the remaining observation time.

### 2.4. Data analysis

#### 2.4.1. Patient selection

**Figure 2:**
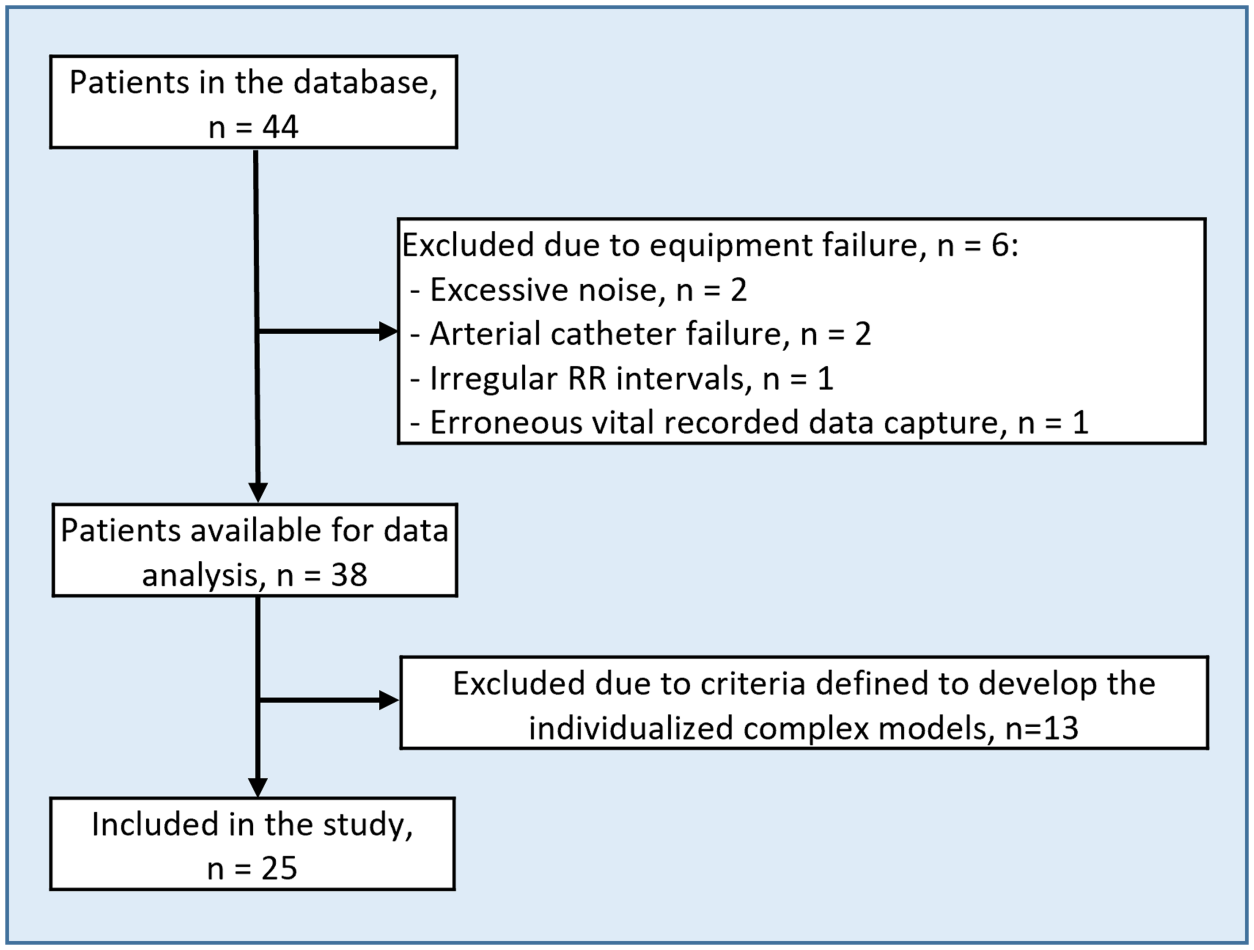
Flow chart of patient selection.

Of 44 patients, 25 were available for the present study after exclusions (Figure 2). Prior to data analysis six patients were excluded due to the following reasons: (1) excessive movement causing the transducer to move relative to the leveling set point and excessive noise (*n* = 2), (2) arterial catheter failure (*n* = 2), (3) irregular RR intervals from pacemaker (*n* = 1) and (4) erroneous vital recorder data capture (*n* = 1). Thus, 38 patients were included in the formal data analysis. Next, the cuffless BP device data was processed to allow for proper training of the complex individualized models and 13 of the 38 patients were excluded because one or more of three criteria were met: (1) Ratio of valid device signals to reference data above 0.6 (*n* = 9), (2) short recordings (total number of reference and cuffless datapoints below 200) (*n* = 11) and (3) to ensure that adequate BP variation was available for the machine learning algorithm, the standard deviation of reference BP in the first half had to be at least 50% of the standard deviation of the reference BP for the whole duration of each individuals data (*n* = 3). Most patients met the criteria related to signal quality and number of reference and device measurement pairs.

#### 2.4.2. Data filtering and processing

Filtering and processing of the data was performed post-hoc in a custom-made database using the Python programming language. Reference BP values were extracted from the raw arterial waveforms. The raw arterial waveform signals were filtered both manually and automatically to reliably remove artefacts from around arterial blood sampling, detachments and re-attachments to the arterial monitoring system, compression of waveforms from wrist flexion, cuff measurements taken at the same arm and high frequency noise. After filtering, reference BP and cuffless BP estimations from the two models were averaged on 15 s epochs. To allow for direct comparison between the two cuffless models, pairwise comparisons between cuffless BP and reference BP were made on the same data in each patient, i.e., the test period defined as the last 50% of data for each patient.

#### 2.4.3. Statistical analyses

Statistical analyses were performed using Stata (StataCorp. 2019. Stata Statistical Software: Release 16. College Station, TX: StataCorp LLC). Data is presented as mean (standard deviation (SD)) or median (interquartile range) if non-normal distribution. We computed mean errors, mean absolute errors (MAE), SD of errors and Bland–Altman plots with bias and 95% limits of agreement (LOA). We are aware that pooling all measurement pairs across all patients may violate the Bland–Altman assumption of independent measurements (11). However, all comparable studies have pooled all measurements in Bland–Altman analyses (12–15). Thus, we chose same methodology for comparative purposes. We also computed Bland–Altman bias and LOA using a proposed method for repeated measures (16) which resulted in bias and LOA (not reported) with negligible differences from the pooled analyses. Correlation analysis was performed using repeated measures correlation as proposed by Bland and Altman (17). In this way the dependency of repeated within subjects are correctly handled. To be able to compare with similar studies, Pearson’s correlation coefficients were also calculated for all measurements across all subjects pooled together.

Comparison of model performance was analyzed in three steps. First, we compared error estimations to determine if they were different from each other. The absolute errors of all measurement pairs (*n* = 7,327) were compared by a non-parametric test for equality of means. Equality of the standard deviation of the errors were compared using a variance comparison test. Second, aggregated BP means per subject from reference BP, the generalized PAT-based model, and the complex individualized model were computed. These means were fitted with the corresponding reference values in a linear regression model for the two models. As these models are not nested, they could not be directly compared by any statistical test. Thus, they were compared numerically on the coefficient of determination (*R*^2^), root mean squared error and Akaike’s and the Bayesian information criterion. Finally, the predictive accuracy of the two models were tested using the Diebold-Mariano predictive accuracy test. The stationary assumption was tested using the augmented Dickey-Fuller test. Sensitivity of the predictive accuracy test, as the stationary assumption may not hold regardless of the result of the augmented Dickey-Fuller test because the data is comprised of different subjects, were tested by performing the Diebold-Mariano test in each subject separately. The overall significance was tested using Fisher’s method of combining *p* values. To test the influence of HR as an additional parameter in the PAT-based model, we also predicted BP using a PAT-only model derived from the data as the PAT and HR-based model. A value of *p* below 0.05 was considered statistically significant.

## 3. Results

**Figure 3:**
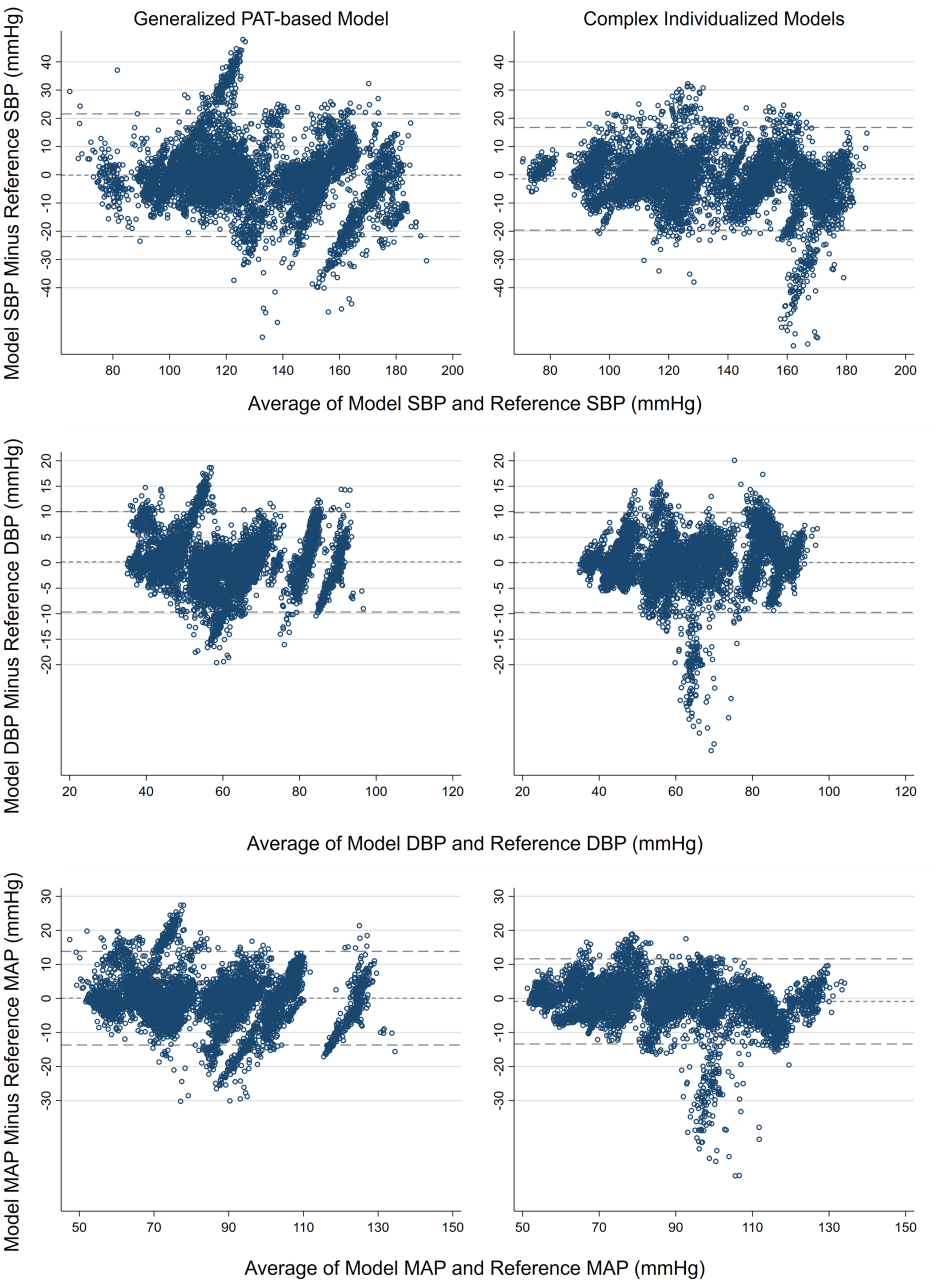
Bland–Altman plots. Mean of reference and model (*x*-axis) plotted against the difference between reference and model (*y*-axis). Horizontal lines indicate bias and upper and lower 95% limits of agreement. SBP, systolic blood pressure. DBP, diastolic blood pressure. MAP, mean arterial pressure.

Patient characteristics are presented in Table 1 and distribution of reference BP across all patients are presented in Table 2. The average number of pairwise comparisons (SD) between reference and the cuffless BP device per subject were 293.2 (161.2), ranging from 124 to 754 with a total of 7,327. Median (Interquartile range) observation time was 4.0 (3.1–4.6) hours with a range from 1.4–8.0 h. Performance of the generalized PAT-based model compared to the complex individualized models are presented in Table 3. The complex individualized models were numerically superior to the generalized PAT-based model across all parameters. Particularly when comparing the repeated measures correlation, more covariation was captured by the complex individualized models compared to the generalized PAT-based model for SBP and MAP where repeated measures correlation coefficients were 0.23 vs. 0.39 and 0.25 vs. 0.37. Results were more similar for DBP compared to SBP and MAP with correlation coefficients of 0.29 (generalized PAT-based model) vs. 0.33 (complex individualized models). Bland–Altman plots with bias and LOA are presented in Figure 3. Bias was close to zero for all BP parameters in both models; −0.2 mmHg vs. −1.4 mmHg, −0.2 vs. 0.0 mmHg and 0.1 mmHg vs. −0.9 mmHg for the generalized PAT-based model vs. the complex individualized models regarding SBP, DBP, and MAP, respectively. LOA favored the complex individualized models for SBP [−21.5, 21.1 mmHg] vs. [−19.2, 16.2 mmHg] and MAP [−13.4, 13.5 mmHg] vs. [−13.9, 11.4 mmHg] but were similar for DBP [−9.8, 9.8 mmHg] vs. [−9.6, 9.6 mmHg]. Percentages of absolute errors within 15, 10 and 5 mmHg (Table 4) also favored the complex individualized models where all percentages were numerically higher for the complex individualized models except for within 15 mmHg regarding DBP. The complex individualized models were significantly different from and outperformed the generalized PAT-based model for SBP and MAP. To the contrary, for DBP, the SD of the errors were not significantly different, and the Diebold-Mariano test of predictive accuracy was not significant. Comparison of the PAT and HR-based model to a PAT-only model showed negligible differences. Pearson’s correlation coefficient and *R*^2^ between the two models were 0.999 and 0.997, respectively.

**Table 1:** Patient characteristics.

Sex, male no (%)	18 (72)
Age, years (SD), range	62.0 (15.4), 27–89
Body mass index, Kg/m^2^(SD)	27.1 (6.4)
Cardiovascular Disease, no (%)	10 (40)
Hypertension, no (%)	17 (68)
Diabetes mellitus type I or II, no (%)	9 (36)
Ongoing intravenous vasopressor treatment, no (%)	2 (8)
Ongoing intravenous vasodilator treatment, no (%)	4 (16)
Ongoing non-invasive continuous or bi-level positive airway pressure, no (%)	2 (8)

**Table 2:** Blood pressure distribution.

	Systolic blood pressure	Diastolic blood pressure	Mean arterial pressure
Mean (SD), mmHg	131.0 (25.7)	61.2 (14.6)	83.9 (18.1)
Range, min-max, mmHg	70.6–194.3	34–100.3	50.9–136.3
Within subject change, median (IQR), mmHg	29.3 (25.0–42.1)	13.4 (12.0–17.0)	18.6 (25.8–27.7)

**Table 3:** Performance of the generalized PAT-based model, the complex individualized models and comparison of the two.

	Generalized PAT-based model	Complex individualized models	*p* value for comparison
**Systolic blood pressure**			
Mean error, mmHg	−0.2	−1.4	
Mean absolute error (SD), mmHg	7.6 (5.3)	6.5 (4.8)	<0.001*
SD of errors, mmHg	7.2	6.7	<0.001**
Median of absolute errors (IQR), mmHg	5.3 (4.5–10.7)	5.8 (4.7–7.3)	
Repeated measures correlation coefficient	0.23	0.39	
Correlation coefficient, all subjects pooled	0.91	0.94	
Linear regression of aggregated data between model and reference***, *R*^2^	0.91	0.96	
Akaike’s information criterion***	173	154	
Bayesian information criterion***	175	156	
Diebold-Mariano comparison of predictive accuracy	Individualized model is significantly better		0.001
**Diastolic blood pressure**			
Mean error, mmHg	0.2	0.0	
Mean absolute error, mean (SD), mmHg	3.3 (3.3)	3.1 (2.2)	<0.001*
SD of errors, mmHg	−3.1	3.0	0.56**
Median of absolute errors (IQR), mmHg	2.7 (1.8–4.1)	2.2 (1.7–3.5)	
Repeated measures correlation coefficient	0.29	0.33	
Correlation coefficient, all subjects pooled.	0.94	0.94	
Linear regression of aggregated data between model and reference***, *R*^2^	0.94	0.94	
Akaike’s information criterion***	131	130	
Bayesian information criterion***	134	133	
Diebold-Mariano comparison of predictive accuracy	Individualized model is non-significantly better		0.14
**Mean arterial pressure**			
Mean error, mmHg	0.1	−0.1	
Mean absolute error, mean (SD), mmHg	4.6 (3.2)	4.0 (2.9)	<0.001*
SD of errors, mmHg	4.4	4.0	<0.001**
Median of absolute errors (IQR), mmHg	3.3 (2.4–6.4)	3.3 (2.5–4.5)	
Repeated measures correlation coefficient	0.25	0.37	
Correlation coefficient, all subjects pooled.	0.93	0.95	
Linear regression of aggregated data between model and reference***, *R*^2^	0.93	0.95	
Akaike’s information criterion***	146	138	
Bayesian information criterion***	149	140	
Diebold-Mariano comparison of predictive accuracy	Individualized model is significantly better		0.006

*Compared using non-parametric test of difference in means of all absolute errors between the two models. **Compared using variance comparison test of equality of standard deviations. ***Means of predicted BP from each model for each subject fitted in a linear regression model against reference BP.

**Table 4:** Percentage of absolute errors within 15, 10, and 5 mmHg.

	Model	Systolic blood pressure	Diastolic blood pressure	Mean arterial pressure
≤5 mmHg	Generalized PAT-based model, %	53.1	78.9	69.2
	Complex individualized models, %	59.2	85.3	78.8
≤10 mmHg	Generalized PAT-based model, %	77.6	96.2	89.6
	Complex individualized models, %	83.8	97	94.2
≤15 mmHg	Generalized PAT-based model, %	87.9	99.7	95.9
	Complex individualized models, %	92.9	98.5	97.8

**Figure 4:**
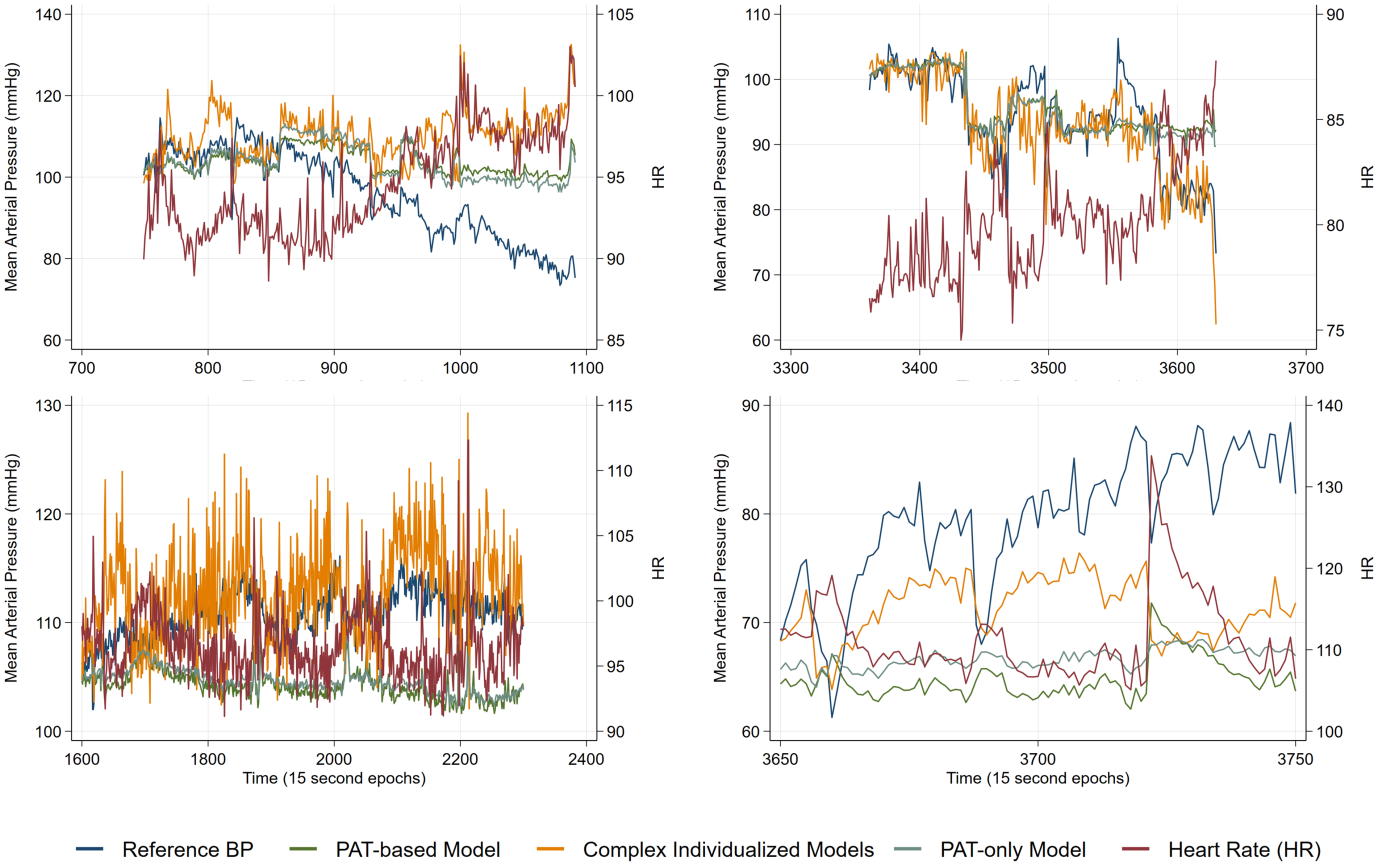
Time series plots from four different patients of reference mean arterial pressure (MAP), heart rate (HR) and predicted MAP from the two models in addition to predicted MAP from a PAT-only model.

An important difference between the generalized PAT-based model and the complex individualized models appeared during the detailed data inspection The generalized PAT-based model performed inadequately in cases of decreasing BP with corresponding heart rate (HR) increase. Therefore, we plotted four different timeseries plots (Figure 4) of four different patients where reduction in BP was coupled with a rise in HR. In the first case (upper left panel) both models were unable to predict the BP reduction, while for the remaining cases, only the complex individualized models correctly predicted the direction of change in BP. Importantly, regarding periods of reduction in BP coupled with a rise in HR, the generalized PAT-based model compared to the PAT-only model showed negligible differences.

## 4. Discussion

Continuous and cuffless non-invasive BP monitoring may improve in-hospital patient monitoring by early detection of clinical deterioration and reduction of adverse outcomes (18). The present study investigated the accuracy of two different predictive BP models using sensor data from a prototype cuffless BP chest belt against intra-arterial measurements in a critically ill ICU cohort. Specifically, we compared a PAT-based model derived from a general population cohort to complex individualized models. The present study had two main findings. First, the generalized PAT-based model did not achieve high accuracy results, indicating that PAT-based BP monitoring in critically ill patients may not be possible, particularly when considering the inability to detect periods of hypotension and tachycardia. Second, the complex individualized models significantly improved accuracy of the cuffless BP device for SBP and MAP, but not DBP, and were able to better track BP changes during hypotension and tachycardia.

The significantly improved accuracy by the complex individualized models sheds light on important challenges regarding non-invasive cuffless BP devices. PAT is frequently cited as a potential non-invasive cuffless surrogate feature in recent years (5). Our results, however, suggests that PAT may not be adequate as cuffless surrogate measurement alone to achieve high accuracy non-invasive BP measurement in critically ill patients. An underlying assumption for general accuracy is stability of the relationship between changes in PAT and changes in BP across individuals, populations and across differing hemodynamic conditions. One or more of these factors likely affect generalizability of PAT as a cuffless surrogate measurement. Several studies have shown that varying between-individuals relationships between PAT and BP are a major limitation (9, 18, 19). The improved accuracy of the complex individualized models indicates that features extracted from ECG and PPG sensors can enable non-invasive cuffless BP monitoring, but these models are patient-specific (and potentially cannot be generalized for all subjects) and rely on machine learning without any *a priori* physiological knowledge. In addition to improved errors, an important finding was the ability of the complex individualized models to better track BP fluctuations, reflected by correlations corrected for repeated within subjects’ measurements (0.23 for the generalized PAT-based model vs. 0.39 for the complex individualized models regarding SBP). It should be kept in mind that correlation across all the data is suppressed by the fact that there were stable periods where BP had low variation.

A concerning finding in our analyses was the inability of the generalized PAT-based model to predict BP changes during some periods of BP reductions coupled with elevation in HR (Figure 4). In our data, the complex individualized models estimated BP better in these situations. In the first scenario in Figure 4 (upper left panel) all models fail, whereas for the next three scenarios the complex individualized models predict the correct direction of BP change while the generalized PAT-based model and the PAT-only model predicts an increase in BP during reduction of reference BP and increases of HR. Our findings suggest that PAT is dependent on HR; an increase in HR causes PAT to decrease independently of the underlying change in BP (a decrease in PAT should always indicate an increase in BP according to the theory). Although conflicting results exists, HR has been shown to affect pulse wave propagation independently of BP similarly to our observations (20, 21). It is also possible that elevated HR is an indication of elevated sympathetic tone, which is shown to increase pulse wave propagation speed independently of central aortic BP (22). This can mask the true BP change in cases were HR and BP change in opposite directions. It should be noted that this was not a pre-specified analysis nor tested in any statistical model, merely, an indication of a potential serious limitation of cuff-based BP monitoring. We interpret this as a need for more data to develop robust models that can accurately estimate BP across differing hemodynamic conditions.

The generalized PAT-based model and complex individualized models achieved LOA of [−21.5, 21.1 mmHg] vs. [−19.2, 16.2 mmHg] regarding SBP and [−13.4, 13.5 mmHg] vs. [−13.9, 11.4 mmHg] regarding MAP. Corresponding results of MAE (SD of errors) were 7.6 (7.2) vs. 6.5 (6.7) and 4.6 (4.4) vs. 4.0 (4.0) regarding SBP and MAP, respectively. These results fall short of accuracy demands required in potentially unstable ICU patients. Particularly when considering the inability of the generalized PAT-based model to predict BP reductions coupled with elevated HR, which is critical in hospitalized patients as such circulatory changes may suggest onset of shock. On the other hand, considering more stable patients and that 78% (generalized PAT-based) and 84% (complex individualized models) of the absolute differences were below 10 mmHg regarding SBP, one may argue that our results are acceptable. It should also be kept in mind that the accuracy of the “gold standard” itself is dependent on appropriate damping as well as leveling and zeroing of the pressure transducer. In everyday management of patients in the ICU, brachial oscillometric cuff BPs are taken regularly. Our LOA were considerably narrower compared to SBP LOA of [−30.2, 31.7 mmHg] revealed in a retrospective analysis comparing oscillometric cuff measurements to invasive measurements in 736 ICU patients (23).

We did not pre-specify any cut-off error statistic because we were evaluating a prototype of the cuffless BP device and the anticipated ISO 81060-3 validation standard applicable to cuffless BP devices was not completed at the time of study planning and data analysis. Acceptance criteria from validation standards aimed at cuff-based devices are not appropriate (24). As a consequence of lack of appropriate validation requirements regarding cuffless BP devices, many have compared against the Association for the Advancement of Medical Instrumentation/European Society of Hypertension/International Organization for Standardization (AAMI/ESH/ISO) criterion; mean error less than 5 mmHg and SD of errors less than 8 mmHg regarding SBP (12, 14, 15). Both our models satisfy this criterion as all mean errors were close to zero. This criterion is, however, intended for standardized cuff measurements seated at rest. Thus, it is difficult to specify clinically accepted accuracy in the study setting. Validation of novel cuffless BP devices dependent on calibration, of which all are at present, should be performed according to the new AAMI/ESH/ISO consensus validation protocol (24). Cuffless BP devices that pass the cuff-intended AAMI/ESH/ISO criterion may not be interpreted as accurate until also passing the new protocol intended to validate initial stability, accuracy during BP changes and reproducibility of stability within the time window of intended use.

Our device performances were comparable to the few similar studies that have investigated accuracy in a cuffless BP device, based on either ECG and PPG or PPG alone, against invasive measurements (12–15). Three of these devices are available on the market (12–14) and one is a prototype (15). It is however difficult to compare results from those directly due to heterogenicity. Our results demonstrated the least narrow LOA compared to SBP LOA of [−10, 10 mmHg] in 10 post cardiac surgery patients (Biobeat wrist watch) (13), [−11.9, 12.2 mmHg] in 23 ICU patients (Aktiia wrist band, PPG) (12), [−11, 16 mmHg] during cardiac catheterization in 17 patients (Senbiosys prototype finger ring, PPG) (15) and [−7.4, 12.8 mmHg] in 20 cardiac ICU patients during controlled short-term supine and in bed measurements (Vitaliti continuous vital signs monitor, ECG and PPG) (14). However, while not achieving as narrow LOA, our study had the most subjects, 25 vs. 10 (Biobeat, ECG and PPG), 23 (Aktiia), 17 (Senbiosys) and 20 (Vitaliti) and by far the largest number of pairwise comparisons of 7,327 compared to 4,000 (Biobeat), 326 (Aktiia), 708 (Senbiosys) and 120 (Vitaliti). Sampling rate also varied between studies from 10 s epochs by Senbiosys to 1-min epochs by Biobeat. All studies excluded a large proportion of patients of which the majority were related to signal selection by algorithms or noise. A particularly important factor regarding cuffless BP devices is the degree of BP change within each patient during data collection. As all devices are dependent on initial calibration, a low change in BP within subjects may result in narrow LOA but the actual ability of these devices to track changes in BP remains unknown. Vitaliti reported measurements only from a stable period immediately following calibration, and Biobeat reported that their subjects were relatively stable as a limitation (within subject ranges not reported). Our subjects had reasonable within subject variations in BP with median SBP (IQR) of 29.3 (25.0–42.1) mmHg with a maximum of 63.2 mmHg. A related issue is reporting of Pearson’s correlation coefficients which are pooled across all subjects, particularly when the devices are calibration dependent and there are repeated measurements within individuals. For comparative purposes we also computed Pearson’s correlation coefficients from all measurements pooled and achieved 0.91 (generalized PAT-based model) and 0.94 (complex individualized models) for SBP compared to 0.94 (Biobeat), 0.87 (Aktiia) and 0.93 (Senbiosys). However, Pearson’s correlation coefficients in this setting does not reflect device accuracy. In contrast, one study found a cuffless BP device using ECG and PPG inaccurate during coronary angiography with SBP LOA of [−2, 70 mmHg] (25). The study was, however, criticized by the manufacturer for incorrect calibration (26).

## 5. Strengths and limitations

A strength in our study is that neither model used any demographic information. The use of demographic information in cuff less research is criticized (27) because demographics itself are known to correlate with BP. Thus, when evaluating accuracy, it is not known how much is related merely to demographics as input in a model. We also provided, to the best of our knowledge, the most datapoints to date in a study evaluating accuracy of a cuffless BP device against invasive arterial measurements. Testing on critically ill patients admitted to an ICU enabled us to reveal the weaknesses of a PAT-based model and the strengths of complex individually fitted models.

We excluded many subjects (43%). However, the majority were related to criteria for developing the complex individualized models and we had comparable proportions and reasons for exclusion to similar studies. Algorithm selection imposes potential limitations on which patients may benefit from cuffless BP in the future. Re-calibration during the data collection in 14 patients may have introduced some overestimation of accuracy. If the device estimation of BP had drifted from reference BP, recalibration would artificially improve error estimates. However, as stated in the methods section, not recalibrating could introduce systemic errors and since the majority only had one recalibration it was decided to recalibrate if the transducer was relevelled. We did not formally test quality of the arterial line by for example the square wave test and calculation of damping coefficients. Since the transducer is levelled on a bracket next to the patient, arterial line BP accuracy is vulnerable to patient movement. We cannot exclude that some variations in reference BP were introduced in this manner. To reliably exclude all periods of which the pressure transducer was out of system, all data collection were observed by an investigator. The critically ill cohort is heterogenous. With a limited number of subjects, we cannot determine which, if any, clinical parameters affected accuracy. PAT can be measured at various places and we are limited to infer our findings to PAT measured at chest level.

## 6. Conclusion

Cuffless BP monitoring is promising, but challenges remain. In the present study, we demonstrated that a generalized PAT-based model measured on the chest did not achieve high accuracy results in critically ill ICU patients and failed to detect clinically important situations. We further demonstrated that more complex and individually fitted models, utilizing more information from the ECG and PPG signals, significantly outperformed the generalized PAT-based model. More data is needed to build robust general models based on machine learning to enable cuffless BP in hospitalized patients.

## Data availability statement

The datasets presented in this article are not readily available because raw signals and data regarding model development may not be disclosed. BP predictions from both models together with reference measurements can be made available upon a formal request. Requests to access the datasets should be directed to sondhe@ous-hf.no.

## Ethics statement

The studies involving human participants were reviewed and approved by REK sør-øst (REC south-east), Oslo, Norway. The patients/participants provided their written informed consent to participate in this study.

## Author contributions

SH, TS, FF, and BW-G contributed to conception and design of the study. SH performed the data collection. KB-R, AS, ØH, and VG organized the database. SH, KB-R, AS, ØH, and VG performed the data analysis and statistical analysis. SH wrote the first draft of the manuscript. All authors contributed to the manuscript revision, read, and approved the submitted version.

## Funding

The research project (Hypersension) was funded by BIA program of the Norwegian research council (project number 332371).

## Conflict of interest

KB-R, AS, and TS were employed by company Aidee Health AS.

The remaining authors declare that the research was conducted in the absence of any commercial or financial relationships that could be construed as a potential conflict of interest.

## Publisher’s note

All claims expressed in this article are solely those of the authors and do not necessarily represent those of their affiliated organizations, or those of the publisher, the editors and the reviewers. Any product that may be evaluated in this article, or claim that may be made by its manufacturer, is not guaranteed or endorsed by the publisher.
